# Preventing Loss of Femoral Periprosthetic Bone Mineral Density in Cementless Total Hip Arthroplasty Using a Tapered Wedge Stem: A Retrospective, Cohort Study in Osteoporotic Patients Treated with Denosumab

**DOI:** 10.7759/cureus.59908

**Published:** 2024-05-08

**Authors:** Keiji Kamo, Hiroaki Kijima, Koichiro Okuyama, Tetsuya Kawano, Nobutoshi Seki, Chie Sato, Tadato Kido, Naohisa Miyakoshi

**Affiliations:** 1 Orthopedic Surgery, Akita Rosai Hospital, Odate, JPN; 2 Orthopedic Surgery, Akita University Graduate School of Medicine, Akita, JPN

**Keywords:** total hip arthroplasty, stress shielding, osteoporosis, denosumab, bone mineral density

## Abstract

Purpose*:* Bone quality is an important issue in elderly osteoporotic patients who undergo total hip arthroplasty (THA) because periprosthetic fracture or aseptic loosening of implant caused by periprosthetic bone loss is a serious concern. Denosumab has been approved for osteoporosis patients. Thus, the purpose of this study was to investigate whether denosumab prevents loss of proximal femoral periprosthetic bone mineral density (BMD) in cementless THA using a tapered wedge stem in patients with osteoporosis.

Methods*:* Seventy consecutive patients who had undergone primary THA were included in this study. Twenty-seven patients who received denosumab for osteoporosis formed the denosumab group, and 43 patients without denosumab formed the control group. Bone turnover markers and femoral periprosthetic BMD were measured at two weeks, six months, and 12 months after THA. BMD was evaluated in seven regions of interest according to the zones of Gruen.

Results*:* BMD in zone 1 was significantly increased from baseline at both six and 12 months after THA in the denosumab group (10.0±10.2%, p<0.001 and 13.1±12.7%, p<0.001, respectively) and significantly decreased in the control group (-3.6±9.7%, p<0.05, and -5.9±9.4%, p<0.001, respectively). BMD in zone 7 was significantly decreased compared to baseline at both six and 12 months after THA in the control group (-19.2±20.2%, p<0.001 and -22.3±16.8%, p<0.001, respectively) but not in the denosumab group (-0.7±18.5% and -1.1±16.6%, respectively). The use of denosumab for THA patients with osteoporosis was independently related to preventing loss of periprosthetic BMD of the femur at 12 months after surgery in zones 1 (p<0.001) and 7 (p<0.001) on multivariate analysis.

Conclusions*:* Denosumab significantly increased proximal femoral periprosthetic BMD in zone 1 and prevented loss of BMD in zone 7 in patients with osteoporosis after cementless THA using a tapered wedge stem at both seven and 12 months. Future studies of denosumab treatment following THA in patients with osteoporosis should focus on clinical outcomes such as the risk of periprosthetic fracture and revision THA.

## Introduction

The proportion of elderly people is rapidly increasing globally. The prevalence of degenerative or traumatic hip disorders requiring surgical intervention is high among elderly populations, so the number of older patients who choose to undergo total hip arthroplasty (THA) to improve their quality of life will presumably increase [1,2]. Although THA is one of the most successful surgeries and provides stable, excellent outcomes for patients, several problems with the procedure remain. For one, bone mineral density (BMD) of the proximal femur around the stem decreases due to stress shielding after cementless THA [3,4].

Female patients with low systemic BMD and high bone resorption marker levels have been reported to show greater periprosthetic proximal femoral bone loss [5]. Severe stress shielding increases the risk of periprosthetic femoral fracture [6-8]. Furthermore, such bone loss can also increase the difficulty of any future revision of THA [7]. Several studies have reported that the loss of BMD in the proximal femur around the stem is reduced by bone antiresorptive therapies [9-13]. However, the preventive effects of bisphosphonates regarding the loss of BMD around the proximal stem are limited [14].

Denosumab is a human monoclonal antibody against receptor activator of nuclear factor kappa-B ligand, the key mediator of the formation, activation, and survival of osteoclasts [15]. In the Fracture Reduction Evaluation of Denosumab in Osteoporosis Every Six Months (FREEDOM) study, denosumab significantly increased BMD as determined by dual X-ray absorptiometry (DXA) not only in trabecular bone but also in cortical bone. This positive effect of denosumab on cortical BMD has not been observed with other antiresorptive therapies for patients with osteoporosis [15]. Denosumab may therefore be more effective than bisphosphonates in reducing the loss of femoral BMD around the stem. However, few reports have examined the preventive effects of denosumab in terms of the loss of femoral periprosthetic BMD in cementless THA for patients with osteoporosis. The purpose of this study was to evaluate the effects of denosumab on femoral periprosthetic BMD after cementless THA using a tapered wedge stem in patients with osteoporosis.

This article was previously posted to the Research Square preprint server on July 28, 2021 (doi: 10.21203/rs.3.rs-736895/v1).

## Materials and methods

**Figure 1:**
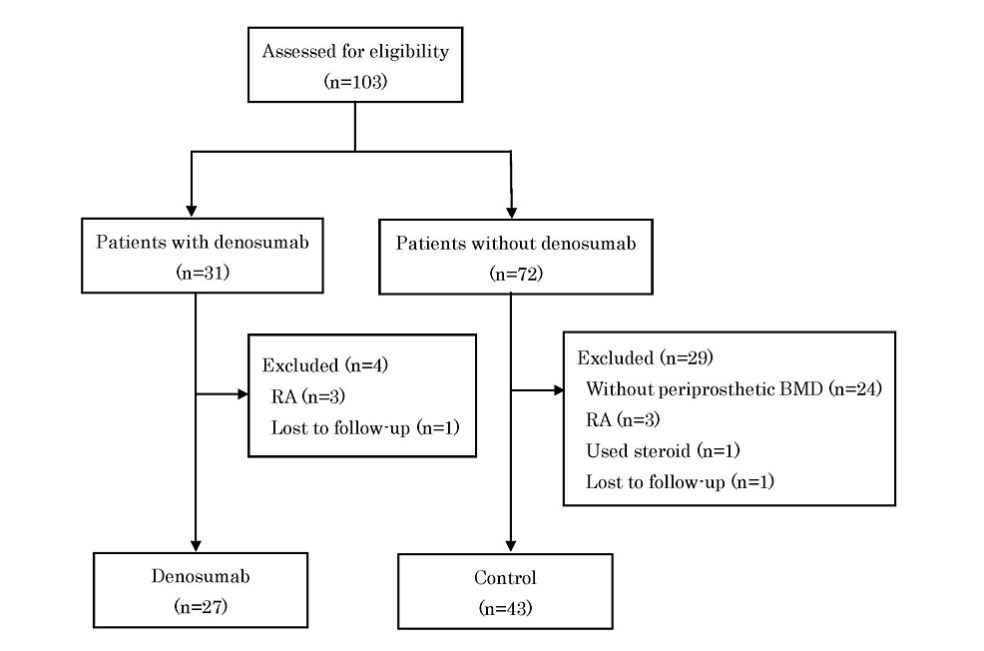
Flowchart of patient selection BMD, bone mineral density; RA, rheumatoid arthritis

This was a retrospective, cohort study. The study was approved by the Institutional Review Board of Akita Rosai Hospital (approval number: 02-03). A total of 103 consecutive patients who had undergone unilateral primary THA using a tapered wedge stem (Anthology, Smith and Nephew, Inc., London, United Kingdom or Accolade II, Stryker Corporation, Kalamazoo, Michigan, United States) under an anterolateral approach in the supine position from May 2015 to May 2021 at Akita Rosai Hospital, Akita, Japan, were retrospectively reviewed (Figure 1). Of these 103 patients, 31 patients with osteoporosis received denosumab before or after THA. All 31 patients who received denosumab fulfilled the Japanese Society of Osteoporosis criteria for the diagnosis of osteoporosis [16]. The exclusion criteria included a history of previous surgery for the ipsilateral hip, evidence of secondary osteoporosis, rheumatoid arthritis (RA), or any other inflammatory arthritides, or treatment with corticosteroids. Three of the 31 patients with osteoporosis who received denosumab were excluded because they had RA, and one patient was lost to follow-up. The denosumab group comprised 27 patients of which one patient received denosumab a year before THA and 26 patients received denosumab from one to three weeks after THA.

The reasons for undergoing THA included osteoarthritis (23 cases) and hip fracture (four cases) in the denosumab group. All patients in the denosumab group received 0.75 µg/day of eldecalcitol to prevent hypocalcemia. The 27 patients with osteoporosis who received denosumab were compared to 43 patients who underwent cementless THA without receiving denosumab at the same institution during the same period. Reasons for undergoing THA in the control group included osteoarthritis (38 cases), osteonecrosis of the femoral head (three cases), and hip fracture (two cases).

**Figure 2:**
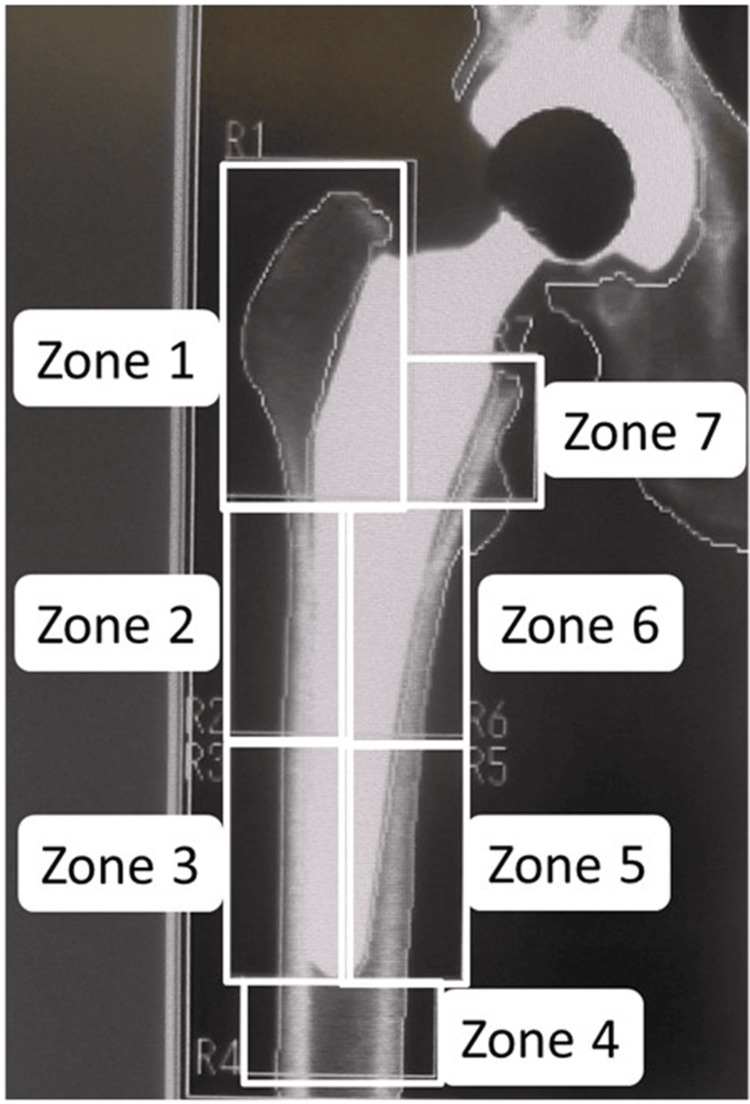
The seven Gruen zones used as regions of interest

All 70 participants were assessed for osteoporosis either preoperatively or at two weeks after THA by measuring BMD at the lumbar spine in the anteroposterior view and determining the presence of a fragility fracture in either the lumbar spine or proximal femur [16]. All patients in the denosumab group and eight patients in the control group met at least one of these criteria for osteoporosis. In the control group, 11 patients were taking medication for osteoporosis before or after THA, with six patients receiving risedronate, three receiving eldecalcitol, one receiving ibandronate, and one receiving a selective estrogen receptor modulator. All patients in this study underwent DXA (QDR-4500SL; Hologic Inc., Marlborough, Massachusetts, United States) to measure femoral periprosthetic BMD at two weeks, six months, and 12 months after THA. BMD measurements around the stem were measured for each of the seven regions of interest described by Gruen (Figure 2) [17]. BMD of the lumbar spine was also measured on the anteroposterior side from L2 to L4 by DXA. Bone turnover markers were measured preoperatively at baseline and then at six months and 12 months after surgery. Bone-type alkaline phosphatase (BAP) was measured as a marker of bone formation, and tartrate-resistant acid phosphatase-5b (TRACP-5b) was measured as a marker of bone resorption. Radiography of the hip joint was performed to evaluate loosening of the femoral stem. The canal flare index (CFI), as described by Noble et al., was also measured preoperatively for the femur on the operative side [18]. CFI was determined as the width of the femoral canal at 20 mm above the mid-trochanteric line divided by the canal width at the isthmus.

Power analysis and statistical analysis

The sample size was calculated using G*Power software (version 3.1.9.7; Heinrich-Heine-Universität Düsseldorf, Düsseldorf, Germany). Effect size, alpha error, and beta error were set as 0.20, 0.05, and 0.20, respectively, based on a previous report [19,20]. The power analysis indicated that a total of 42 samples (21 per arm) would be needed.

To compare the baseline characteristics of patients in the two groups, the chi-squared test was used for comparisons of categorical variables, whereas the Mann-Whitney U test was used for continuous variables. Mean percentage changes in BMD around the stem and of bone turnover markers (BAP, TRACP-5b) were evaluated in each of the two groups using Friedman’s test. Post-hoc comparisons were performed using Bonferroni correction for multiple comparisons. Multivariate analysis was also performed to identify factors related to BMD preservation at the proximal femur in zones 1 and 7 at 12 months after THA using six variables (age, body mass index, lumbar BMD, type of stem, CFI, and use of denosumab). All statistical analyses were performed using R software (version 3.6.3; R Foundation for Statistical Computing, Vienna, Austria). Probability values of p<0.05 were considered significant.

## Results

Baseline demographic and clinical characteristics are shown in Table 1. The mean age at baseline was 74.0 years in the denosumab group and 70.8 years in the control group. Baseline BMI and lumbar spine BMD were significantly lower in the denosumab group than in the control group. No significant differences in type of stem, concentrations of bone turnover markers, or CFI were evident between the two groups.

**Table 1:** Baseline characteristics of patients Continuous values are given as mean ± standard deviation. Categorical values are given as numbers. BMI, body mass index; BMD, bone mineral density; DSM, denosumab; RIS, risedronate; Eld, eldecalcitol; IBA, ibandronate; SERM, selective estrogen receptor modulator; BAP, bone-type alkaline phosphatase; TRACP-5b, tartrate-resistant acid phosphatase-5b

Characteristic	Denosumab (n=27)	Control (n=43)	P value
Age (years)	74.0±9.4	70.8±10.3	0.23
Range (years)	58–91	48–86	
Sex (male : female)	1 : 26	5 : 38	0.64
BMI (kg/m^2^)	22.2±3.1	26.1±4.7	<0.01
Type of stem (Anthology : Accolade II)	10 : 17	22 : 21	0.22
Osteoporosis (n)	27	8	
Drug for osteoporosis	DSM n=27	RIS n=6, Eld n=3, IBA n=1, SERM n=1	
Lumbar spine BMD (g/cm^2^)	0.76±0.13	0.98±0.18	<0.001
BAP (µg/l)	15.6±5.6	16.8±5.8	0.32
TRACP-5b (mU/dl)	553±171	549±211	0.46
Canal flare index	3.42±0.59	3.59±0.67	0.33

**Figure 3:**
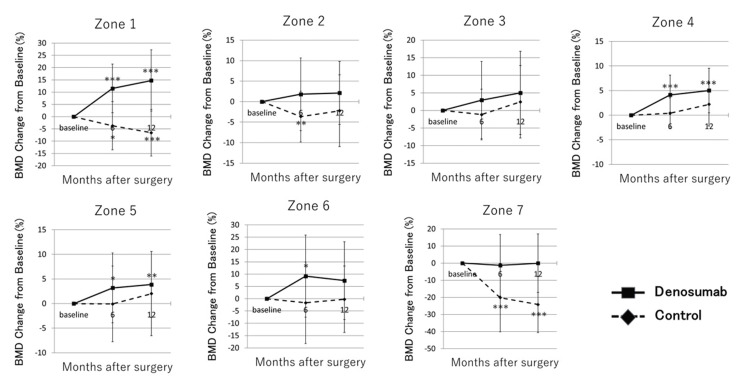
Mean percentage change in BMD around the femoral tapered wedge stem *p<0.05 vs. baseline, **p<0.01 vs. baseline, ***p<0.001 vs. baseline BMD, bone mineral density

**Figure 4:**
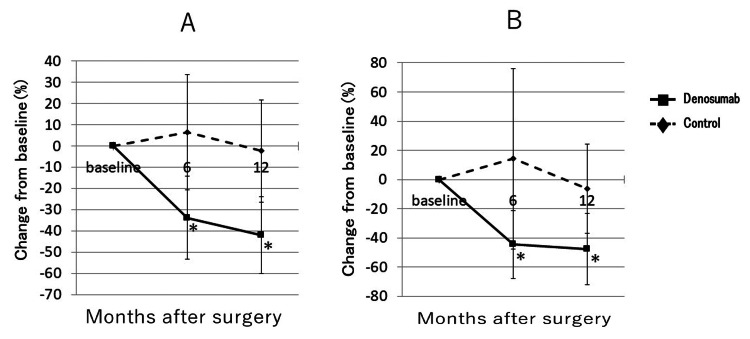
Mean percentage changes in bone turnover markers (A) Bone-type alkaline phosphatase (BAP); (B) Tartrate-resistant acid phosphatase-5b (TRACP-5bB) *p<0.001 vs. baseline

Figure 3 shows the mean percentage change in BMD around the femoral stem according to Gruen zones. The mean percentage change to BMD in zone 1 was significantly higher at both six and 12 months after THA than at baseline in the denosumab group (10.0±10.2%, p<0.001 and 13.1±12.7%, p<0.001, respectively), but significantly lower in the control group (-3.6±9.7%, p<0.05, and -5.9±9.4%, p<0.001, respectively). A significant decrease to BMD in zone 7 compared to baseline was seen at both six and 12 months in the control group (-19.2±20.2%, p<0.001 and -22.3±16.8%, p<0.001, respectively), but not in the denosumab group (-0.7±18.5% and -1.1±16.6%, respectively). Figure 4 shows the mean percentage change in bone turnover markers. BAP was significantly lower at six and 12 months after surgery than at baseline in the denosumab group (-33.1±19.4%, p<0.001 and -39.9 ±18.8%, p<0.001, respectively). TRACP-5b was also significantly lower at six and 12 months after THA than at baseline in the denosumab group (-44.9±23.8%, p<0.001 and -46.8 ±25.5%, p<0.001, respectively). No significant differences in BAP were identified in the control group. TRACP-5b was significantly lower at 12 months after THA than at baseline in the control group (-8.7±28.0, p<0.05). On radiographic evaluation of the hip joint, no stem loosening was identified in any patients in either the denosumab or control group at six and 12 months after THA. On multivariate analysis, use of denosumab was independently associated with change in periprosthetic BMD at the proximal femur in zone 1 (B=19.5, 95%CI 12.7-26.3, p<0.001; adjusted R2=0.393) (Table 2) and zone 7 (B=22.0, 95%CI 11.7-32.2, p<0.001; adjusted R2=0.287) (Table 3) at 12 months after THA. In zone 7, CFI also correlated with change in periprosthetic BMD (B=8.16, 95%CI 1.31-15.0, p<0.05) at 12 months after THA (Table 3).

**Table 2:** Multiple regression analysis for factors affecting percentage change in BMD for Zone 1 at 12 months after THA BMI, body mass index; BMD, bone mineral density; CFI, canal flare index; THA, total hip arthroplasty

Factor	Regression coefficient	95% confidence interval	P value	R²
Model summary			<0.001	0.393
Age	0.08	-0.21 to 0.38	0.58	
BMI	-0.17	-0.91 to 0.57	0.65	
Lumbar BMD	8.04	-10.1 to 26.1	0.38	
Type of stem	3.53	-2.21 to 9.28	0.22	
CFI	0.36	-4.22 to 4.94	0.88	
Use of denosumab	19.5	12.7 to 26.3	<0.001	

**Table 3:** Multiple regression analysis for factors affecting percentage BMD change in Zone 7 at 12 months after THA BMI, body mass index; BMD, bone mineral density; CFI, canal flare index; THA, total hip arthroplasty

Factor	Regression coefficient	95% confidence interval	P value	R²
Model summary			<0.001	0.287
Age	0.08	-0.36 to 0.53	0.71	
BMI	-0.41	-1.52 to 0.69	0.46	
Lumbar BMD	5.37	-21.7 to 32.5	0.69	
Type of stem	0.15	-8.45 to 8.76	0.97	
CFI	8.16	1.31 to 15.0	<0.05	
Use of denosumab	22.0	11.7 to 32.2	<0.001	

## Discussion

The present study demonstrated preventive effects from denosumab on loss of femoral periprosthetic BMD at 12 months after surgery in zones 1 and 7 after cementless THA using a tapered wedge stem among patients with osteoporosis. Moreover, BMD in zone 1 was significantly increased from baseline at both six and 12 months after THA in the denosumab group. This result was consistent with a previous study using a tapered wedge uncemented stem [19]. Aro et al. showed that denosumab significantly decreased bone loss in the femoral neck (zone 7) and increased periprosthetic BMD in the greater trochanteric region (zone 1) [19]. Nagoya et al. reported that denosumab increased the percentage change in periprosthetic BMD at zone 7 by an average of 7.3%, but did not increase at BMD in zone 1 among patients with cementless THA using a Zweymüller-type stem [13]. This discrepancy was attributed to the differences between the tapered wedge stem and Zweymüller-type stem.

Periprosthetic BMD of the proximal femoral stem was significantly decreased in zone 7 even with the use of bisphosphonates, although bisphosphonates prevented some loss of proximal periprosthetic BMD in zones 1 and 7 compared to the control group not given bisphosphonates [9,11,21]. In the present study using denosumab, no significant decrease in BMD was seen in zone 7 compared to baseline after THA, and denosumab may thus be more effective for preventing periprosthetic BMD in the proximal femur than bisphosphonates. In addition, multivariate analysis in this study showed that the use of denosumab was independently associated with changes in periprosthetic BMD of the proximal femur in zones 1 and 7. Poole et al. reported that denosumab significantly increased cortical mass surface density and thickness at the proximal femur among women with osteoporosis [22]. These positive effects of denosumab on cortical bone in the proximal femur are not seen with bisphosphonates. The antiresorptive effects of bisphosphonates are not sufficient in cortical bone, because concentrations of bisphosphonates are lower in cortical bone than in trabecular bone [23,24]. In contrast, denosumab circulates freely to bone surfaces and into remodeling compartments, inhibiting osteoclastogenesis and thus inhibiting remodeling more rapidly and more markedly than alendronate in cortical bone [24]. Since the proximal femur shows greater cortical bone mass than cancellous bone mass after cementless THA using a tapered wedge stem, use of denosumab, which has marked effects on cortical bone, is considered effective for maintaining periprosthetic BMD. In addition, Zebaze et al. reported that denosumab reduced the cortical porosity of the proximal femoral shaft, resulting in increased mineralized matrix volume and improved strength [25]. These effects of denosumab may reduce the risk of periprosthetic femoral fractures after cementless THA.

Several limitations to this study need to be kept in mind when interpreting the results. First, the present study included only a small sample and was retrospective in design. Further randomized or prospective studies of larger cohorts are needed to investigate the preventive effects of denosumab on the loss of femoral periprosthetic BMD using a tapered wedge stem in patients with osteoporosis. Second, the present study had a short-term follow-up period. BMD in the calcar region continued to decrease faster than would be expected from normal aging up to 14 years after THA using a tapered uncemented stem [26]. However, BMD has been reported to decrease markedly in the proximal femur during the first year postoperatively and bone loss was minimal, at only a few percentage points per year after the first postoperative year [27]. Thus, preventing loss of BMD in the proximal femur around the stem is important within the first 12 months after THA. Third, some patients in the control group were taking medications for osteoporosis, which may have affected the study results. However, denosumab has been shown to be effective in preventing BMD loss in the proximal femur, even though some patients in the control group may have obtained positive effects from drugs they were taking for osteoporosis in the present study.

## Conclusions

Denosumab significantly increased periprosthetic BMD in zone 1 and prevented loss of BMD in zone 7 of the proximal femur among patients with osteoporosis after cementless THA using a tapered wedge stem at both six and 12 months after surgery. The use of denosumab for THA patients with osteoporosis was independently related to preventing the loss of periprosthetic BMD in zones 1 and 7 of the proximal femur on multivariate analysis. Future studies of denosumab treatment following THA in patients with osteoporosis should focus on clinical outcomes such as the risks of periprosthetic fracture and revision THA.
